# Gut Microbiome and Its Role in Valvular Heart Disease: Not a “Gutted” Relationship

**DOI:** 10.3390/life14040527

**Published:** 2024-04-19

**Authors:** Gyanaranjan Nayak, Kyriakos Dimitriadis, Nikolaos Pyrpyris, Magdalini Manti, Nikolaos Kamperidis, Vasileios Kamperidis, Antonios Ziakas, Konstantinos Tsioufis

**Affiliations:** 1 First Department of Cardiology, School of Medicine, National and Kapodistrian University of Athens, Hippokration General Hospital, 115 27 Athens, Greece; drgyana10@gmail.com (G.N.); npyrpyris@gmail.com (N.P.); ktsioufis@gmail.com (K.T.); 2 St Mark’s Hospital, Imperial College London, London HA1 3UJ, UKnkamperidis@yahoo.com (N.K.); 3 First Cardiology Department, AHEPA University Hospital, Medical School, Aristotle University of Thessaloniki, 54453 Thessaloniki, Greece; vkamperidis@outlook.com (V.K.); tonyziakas@hotmail.com (A.Z.)

**Keywords:** cardiovascular disease, valvular heart disease, gut microbiome, inflammation, oral microbiome

## Abstract

The role of the gut microbiome (GM) and oral microbiome (OM) in cardiovascular disease (CVD) has been increasingly being understood in recent years. It is well known that GM is a risk factor for various CVD phenotypes, including hypertension, dyslipidemia, heart failure and atrial fibrillation. However, its role in valvular heart disease (VHD) is less well understood. Research shows that, direct, microbe-mediated and indirect, metabolite-mediated damage as a result of gut dysbiosis and environmental factors results in a subclinical, chronic, systemic inflammatory state, which promotes inflammatory cell infiltration in heart valves and subsequently, via pro-inflammatory molecules, initiates a cascade of reaction, resulting in valve calcification, fibrosis and dysfunction. This relationship between GM and VHD adds a pathophysiological link to the pathogenesis of VHD, which can be aimed therapeutically, in order to prevent or regress any risk for valvular pathologies. Therapeutic interventions include dietary modifications and lifestyle interventions, in order to influence environmental factors that can promote gut dysbiosis. Furthermore, the combination of probiotics and prebiotics, as well as fecal m transplantation and targeted treatment with inducers or inhibitors of microbial enzymes have showed promising results in animal and/or clinical studies, with the potential to reduce the inflammatory state and restore the normal gut flora in patients. This review, thus, is going to discuss the pathophysiological links behind the relationship of GM, CVD and VHD, as well as explore the recent data regarding the effect of GM-altering treatment in CVD, cardiac function and systemic inflammation.

## 1. Introduction

Cardiovascular disease (CVD) is the leading cause of morbidity and mortality worldwide [1]. While CVD is well associated with traditional risk factors like hypertension, hyperlipidemia, smoking, diabetes and metabolic syndrome, with a plethora of data describing their relationship, recent research and microbial sequencing analysis has shown the significance of the gut microbiome (GM) in affecting cardiovascular physiology and promoting pathogenetic mechanisms, ultimately responsible for the manifestation of cardiovascular pathologies [2,3]. The GM can be defined as the entirety of microbial organisms, ranging from bacteria to eukaryote and archea, populating the gastrointestinal tract, estimated to be around 10^14^ and possibly overly excessive compared to human cells in both number and genomic content [4]. Along with the GM, oral microbiota (OM) is an increasingly recognized microbiome site, that consists of the normal microbiome present at the oral cavity of each individual and is one of the largest and most complex microbiomes in the human body [5]. The GM seemingly affects the entire spectrum of CVD, and recently, novel research evaluated its role, including OM, in the pathogenesis and progression of valvular heart disease (VHD). Both the GM and OM seem to be related to VHD similarly to other CVD, as a risk factor promoting inflammation and altered host metabolism. This review aims to delve into the role of the GM and OM in the pathophysiology of VHD and describe available and emerging therapeutic options, targeted towards restoring the normal gut flora, including up-to-date animal and clinical evidence.

## 2. Pathophysiology: The Gut and Heart connection

### 2.1. Gut Microbiome: From Physiology to Pathogenesis

The GM predominantly inhabits the colon and is mostly anaerobic. Recent genomic analyses indicate that a large number of phyla colonize the human gut, with the majority being *Proteobacteria*, *Firmicutes*, *Actinobacteria and Bacteroidetes* [6]. The colon is most probably colonized after birth, despite conflicting evidence regarding the presence of a placental microbiome [7,8], and it rapidly increases and alters in the early stages of life, as a result of environmental factors such as diet, antibiotic use, disease and type of delivery (vaginal or c-section) [9,10]. Its symbiosis with human hosts comes with a number of benefits for human physiology, including integrity of the gastrointestinal mucosal barrier, vitamin and nutrients’ metabolism and protection against pathogens [11]. Of note, the GM can alter cell genomic expression cells via producing short chain fatty acids (SCFAs) and limit bacterial translocation [12,13], while also influencing epithelial homeostasis [14] and regulating both intestinal mucosal and systemic immune systems [15]. However, the positive symbiotic effects can be diminished or even reversed in case of dysbiosis development. Dysbiosis, an imbalance between the host and GM, has been already described to contribute to multiple pathologies, including autoimmune disease [16], thyroid disease [17], COVID-19 and CVD [18,19].

Pathophysiologically, GM-mediated CVD implications are complex and can be categorized as either direct, microbe-mediated or indirect, metabolite-related. In respect to the damage caused by microbial dysbiosis, microbe-induced systemic inflammation can promote CVD pathogenesis. This is particularly notable when examining the gut bacteria-derived lipopolysaccharide (LPS), which is normally produced by gram-negative intestinal bacteria and can be in increased serum concentrations following loss of intestinal cell integrity, facilitated via either local dysbiosis and LPS-mediated damage to the epithelial barrier [20] or other pathologies leading to disrupted intestinal blood vessel molecule transfer, such as hypertension [21]. The accumulation of LPS in the human body results in low-grade chronic inflammation, which is present in atherosclerotic, but not normal arteries [22]. In particular, a proposed mechanism concerns LPS binding to toll-like receptor 4 (TLR4), and a subsequent systemic inflammatory reaction mediated by the secretion of pro-inflammatory molecules and enhancement of pro-atherogenic receptors, as shown by studies linking receptors and molecules commonly recognized in atherosclerotic plaques and endothelial dysfunction phenotypes [23]. LPS levels and chronic inflammation have been extensively studied, with evidence of its role in atherosclerosis [24], atrial fibrillation [25] and heart failure [26]. It is of note that mutations in TLR4, leading to lack of binding of LPS to the receptor, may be related with a lower atherogenic risk, but not inflammation burden [27]. Furthermore, gut microbe-secreted metabolites such as trimethylamine N-oxide (TMAO), bile acids and SCFAs may alter the course of CVD. These molecules are linked to increased inflammatory states via various complex pathways (i.e., mitogen-activated protein kinase, extracellular signal-related kinase, and the nuclear factor-κB (nf-Κb) pathway) [28], as well as non-inflammation-dependent pathogenetic mechanisms including thrombus formation, atherogenesis, fibrosis and foam cell formation [29,30], and may be also related to increased major adverse cardiovascular events [31]. It is of interest that the adverse effects of such molecules are exacerbated, regarding both cardiac function and fibrosis, when dietary factors promoting the formation of such molecules (TMAO, choline) are given to mice models [32,33], while partial inhibition of these molecules may result in enhanced cardiac [34] and renal [35] function. On the other hand, the role of SCFAs may be more protective, limiting inflammation, metabolic disorders and atherogenesis [36]. Therefore, absence of SCFAs formatting bacteria in a dysbiotic environment, which promotes the secretion of the aforementioned metabolites, may lead to loss of this protective effect and thus to CVD. Finally, other recently recognized metabolites of gut microbiome, such as phenylacetylglutamine, may have an inflammation-independent role in the pathogenesis of CVD. In specific, Liu et al. [37] showed that in patients with suspected coronary artery disease (CAD) undergoing computed tomography coronary angiography, patients with increased levels of phenylacetylglutamine had significantly increased rates of obstructive CAD, high-complexity lesion and high-risk plaque phenotype, as well as adrenergic receptor activation and increased platelet activation.

### 2.2. GM and VHD

**Figure 1:**
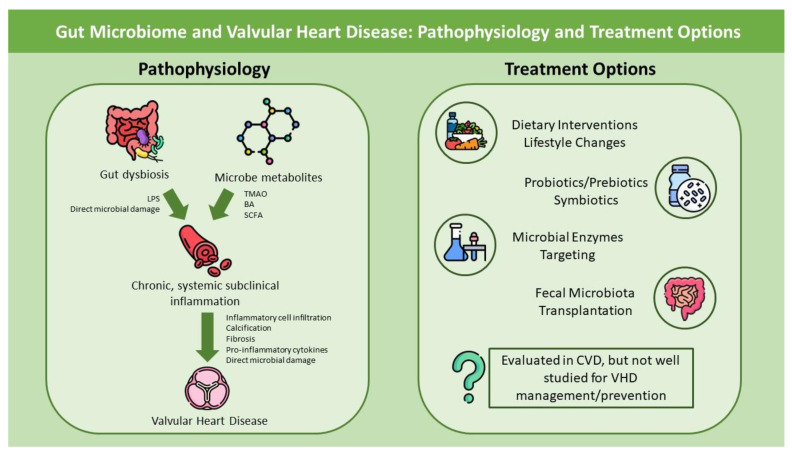
The role of the gut microbiome in the pathophysiology of valvular heart disease and treatment considerations. Abbreviations: LPS: lipopolysaccharides; TMAO: trimethylamine N-oxide; BA: bile acids; SCFAs: short chain fatty acids; CVD: cardiovascular disease; VHD: valvular heart disease.

Recent studies have highlighted that, along with CAD, GM dysbiosis may also be related to VHD, as the pathobiology of CAD and specific VHD, such as aortic stenosis (AS), has many similarities [38]. As aforementioned, the most probable pathogenetic mechanisms come as a result of pathogen-mediated (dysbiosis) or metabolite-related mechanisms, both resulting in an altered host inflammatory state, which promotes calcification and structural valve dysfunction (Figure 1). In this setting, some researchers evaluated the role of GM in VHD (Table 1). Curini et al., reported the first taxonomical and functional characterization of human calcific aortic stenosis with the associated microbiota [39]. In 20 patients with severe symptomatic calcific AS, infiltration of T cells, and specific T-helper cells, was present in all patients, in response to microbe presence detected by ribonucleic acid (RNA) sequencing. Furthermore, CD8+ cells were found significantly increased in a proportion of German patients, compared to Italians. The presence of chronic inflammation, in response to microbiota presence, and the ability of T-cells to mediate altered calcium metabolism and valve calcification provide a hint for the potential role of GM in the pathogenesis of AS [40]. Furthermore, the study showed that the most prevalent phylum was *Bacteroidota*, followed by *Proteobacteria* and *Firmicutes*. *Proteobacteria* have also been found in specimen analysis of mitral valves. Thus, these studies are hypothesis-generating regarding the type and role of the microbiome, via initiating an inflammatory response, in VHD and valve calcification.

The relationship of AS and GM has also been explored in terms of evaluating the role of GM-derived metabolites and the presence or severity of AS. Kocyigit et al., showed that choline levels were significantly increased in patients with severe AS; however, they did not find a similar association with TMAO levels. It is of interest that this study revealed that higher choline levels are associated with higher aortic and mitral annular calcification scores, while its levels were significantly more elevated in patients with more dense lymphocyte infiltration, osseous metaplasia and calcification in the aortic valve [41], thus relating choline levels with both AS presence and severity. Similar results for choline levels and VHD have also been showcased by other investigators [42], revealing a significant relationship. TMAO has also been linked to aortic stenosis, with Guo et al. [43], evaluating patients with severe aortic stenosis, reporting significantly higher TMAO levels in patients with versus without aortic stenosis, even after adjusting for confounders. Furthermore, increased TMAO levels were predictive of patient survival, as they were also significantly associated with 2-year all cause and late cumulative mortality, while being an independent mortality predictor in multivariate analysis [43]. Finally, a recent study by Xiong et al., examining the role of TMAO in valvular fibrosis in human aortic valve interstitial cells treated with TMAO, showed that it is significantly related to aortic valve fibrosis, and specifically by initiating endoplasmic reticulum stress mechanisms involving activation of PERK/ATF-4 and IRE-1α/XBP-1s pathways. Treatment with 3,3-dimethyl-1-butanol, which inhibits the formation of TMAO, leads to reduced fibrosis. Finally, high-choline and fat diet in treated mice was shown to increase TMAO levels and activation of the aforementioned pathways, thus subsequently leading to increased fibrosis [44].

Lastly, it should be mentioned that, despite the initial link to CAD, studies revealed that it is probable that different bacteria populations have a distinctive role in each pathogenetic process [45]. In more detail, in a recent study, the CAD cohort was predominantly populated by *Collinsella aerofaciens*, *Enterococcus*, *Megamonas* and *Megasphaera*, while the VHD by *Bacteroides plebeius*, *Enterobacteriaceae*, *Veillonella dispar* and *Prevotella copri*. Interestingly, *Blautia*, a bacteria linked with anti-inflammatory response and producing short-chain fatty acid (SCFA) was reduced. The correlation analysis reported that for VHD *Prevotela corpi* and for CAD *Collinsella aerofaciens* may be key in the pathogenetic process related to the GM. This study, along with showcasing the different phenotypes of GM in patients with different CVD, also promotes a more inflammation-driven pathophysiological mechanism for VHD, as shown by the properties of their flora, in comparison to multiple different mechanisms, including promotion of dyslipidemia and metabolic syndrome in CAD patients. In other words, despite the vast of similarities between the pathophysiology of CAD, CVD in general and VHD, it is more likely that GM, its metabolites and the subsequent inflammation actively act and influence the physiology of the valvular mechanism, and possibly act concomitantly with other CVD risk factors, in order to contribute to each pathology. However, such conclusions could not be definitively drawn from these results.

### 2.3. OM, CVD and VHD

The role of OM in VHD is also well known, when considering the large number of endocarditis events following dental interventions; however, on top of acute infections, evidence suggest that periodontitis, oral dysbiosis and transient bacteremia with associated low-grade inflammation can modulate host inflammatory response and be linked to CVD [46]. Periodontitis has already been linked to atherosclerosis, with analyses showcasing inflammation as the responsible mediator for promoting atherogenesis [47]. Early studies show that the presence of at least one oral bacterium is frequent (44%) in atherosclerotic plaques [48]. Recently, analyses showed that bacteria such as *Porphyromonas gingivalis*, which are present in periodontitis, can also be identified in atheromas and are associated with the activation of the NF-κB-BMAL1-NF-κB signaling loop [49], while there might be a relation of periodontitis with myocardial infarction and major adverse cardiovascular events [50]. Nevertheless, a consensus document recognizes the increased risk for CVD in patients with periodontitis and the potential pathophysiological relationship between the two pathologies. However, given the common risk factors for periodontitis, chronic subclinical inflammation and atherosclerosis, establishing a causal relation cannot be entirely confirmed until more definitive data are available. Similarly, there are very limited data specifically addressing non-infectious, inflammation-mediated damage to heart valves mediated by OM. Older, specimen studies have identified oral pathogens in cardiac specimens, including cardiac valves, reporting high rates of *Streptococcus mutans* as well as low rates of periodontitis-related microbes [51,52]. Sia et al., recently showed that the incidence of VHD is significantly more frequent in patients with periodontitis, compared to controls, with periodontitis being independently related to the development of VHD. Interestingly, treatment for periodontitis was associated with a significantly lower incidence of VHD [53]. However, as the study found differences between the commonly identified oral pathogens present at atherosclerotic plaques (*Porphyromonas gingivalis*) with those populating valves (Group A Streptococci), the investigators mention that a conclusion regarding whether valve damage is mediated by chronic inflammation or subclinical infective endocarditis cannot be made and this topic warrants more research. Such pathophysiological hypotheses have not yet been elaborately studied and thus represent a frontier of research, in order to better understand potential links between VHD and OM beyond infective endocarditis-mediated damage.

### 2.4. Associations with VHD Management

The role of GM in VHD is not only pathogenetic but can also complicate the course of its management. Antibiotic use can alter the normal GM of a patient undergoing cardiac surgery, with studies showing decreased levels of beneficial bacteria and increased levels of harmful bacteria, such as *Enterococcus*, in post-operative cardiac patients [54]. The link between such disruptions and adverse events in operated patients is, however, still undetermined. Similarly, Xue et al., also showed that IV antibiotics influence GM composition in patients undergoing cardiac surgery, predominantly those undergoing valve replacement, with 7-day administration being able to entirely disrupt gut–host symbiosis [55]. Mostly, antibiotics with biliary excretion were responsible for such changes. Notably, such differentiations in the GM may also have implications in anticoagulation treatment, especially with vitamin K antagonists (VKA). Vitamin K is predominantly produced by Gram-positive bacteria in the gastrointestinal tract [56], this IV antibiotic excreted via the biliary can negatively influence vitamin K synthesis and consequently treatment with VKAs. More recently, similar investigations were performed regarding the role of gut dysbiosis, VKA and direct oral anticoagulation (DOAC) in rat models. The investigators reported that in antibiotic-treated rats, there are changes in the microbiome that affect oral anticoagulant (OAC) metabolism. In specific, warfarin and rivaroxaban had increased bioavailability, contrary to dabigatran, which showed a decreased bioavailability. Confirming these results, the study also showed altered expression of hepatic enzymes responsible for OAC metabolism, including p-glycoprotein, the nuclear receptor PRX and CYP1A2, CYP2C9 and CYP3A3, in animals treated with antibiotics compared to controls [57]. The aforementioned results highlight the significant, yet understudied, role of GM in the whole spectrum of VHD, from pathophysiology to management, and indicate cautious management of medication in patients with suspected gut dysbiosis, as it could lead to suboptimal patient outcomes and OAC failure.

## 3. Therapeutic Approaches to Restore the Normal Gut Flora

As elaborately described, the GM and OM have a distinctive pathophysiological relationship with CVD and VHD. Therefore, targeting gut dysbiosis and metabolites of the GM, in order to diminish their harmful impact, could be a novel therapeutic target for CVD. There are, currently, several options aiming to restore normal gut flora, including non-pharmacological, pharmacological and interventional (Figure 1).

### 3.1. Lifestyle Interventions: Diet and Oral Hygiene

A well-balanced diet is a key pillar to disease prevention, regardless of its association with the GM. However, its benefits may extend to maintaining a normal gut flora or restoring gut dysbiosis. It is of note that diet can play a significant role in the GM phenotype, dysbiosis and VHD risk. Specifically, Curini et al. [39] showed that in patient groups from different countries (Italy and Germany), there are significant differences in the microbes present in their valves, with Germans having more CVD-specific microbial infiltrates. Given the presumable differences in diet between the two populations (typical, meat and high-fat diet for Germans and Mediterranean diet for Italians), it could be possible that such dietary choices, promoting formation of GM-associated metabolites such TMAO, could promote valvular damage mediated by the GM. It is well known that diet has an incremental role in the GM, and a high-fat diet can alter its synthesis and promote dysbiosis even in extremely short periods of time [58], while it can also be related with increased LPS and levels of inflammatory markers [59]. Furthermore, such diets can diminish the production of the protective SCFAs in human organisms, which sustains the vicious cycle of GM dysbiosis, chronic inflammation and disease [60]. On the other hand, a diet with fibers can be beneficial, as fibers are known to promote normal gut flora, maintain GM diversity and promote an optimal GM–host relation [61]. Therefore, following a well-balanced diet, such as the Mediterranean diet, which has a well-documented protective effect in the cardiovascular system, can be of benefit in such individuals, by promoting microbial diversity, enhancement of SCFA-producing species, reduction of harmful metabolite production and decrease gut permeability [62,63]. This has also been shown in large, randomized trials examining the effect of different diets in secondary prevention of CVD, such as the CORDIOPREV study, where the Mediterranean diet was found to be superior to a low-fat diet in the prevention of major adverse cardiovascular events in patients with established CAD [64]. Notably, this is particularly significant in special populations, such as athletes, where a well-balanced diet, like the Mediterranean, is not usually followed. The effect of daily dietary intake aimed to optimize athletic outcomes, as opposed to the regular diet followed by the general population, may also have an effect in their cardiovascular health, in a similar manner different exercise modalities has [65]. Future research should examine such parameters of dietary options, specifically focusing on such populations, in order to identify possible links with cardiac function alterations and CVD.

Regarding the role of oral dysbiosis and periodontitis, diet is also a risk factor for its development, with a high-fat and sugar and low-fiber diet being well associated with periodontitis [66]. Therefore, similarly to the GM, diet intervention should be initiated in those patients, with the aim of following dietary plans rich in omega-3 fatty acids, vitamins and fibers and low in fat and carbohydrates, which have been shown to reduce periodontal inflammation [67]. Furthermore, diets including the Mediterranean and Dietary Approaches to Stop Hypertension (DASH) are also beneficial in reducing the risk of periodontal pathologies, and should be used, especially in accordance with patient phenotype, such as DASH for patients with hypertension [68]. Regarding oral hygiene, it has been shown that its suboptimal implementation in adults can increase the risk of periodontitis by up to five times, while good oral hygiene is associated with low rates of such complications [69]. Therefore, in accordance with advice from dentists, individuals should follow oral hygiene rules, with frequent toothbrushing and flossing, as well as visits to dental experts, in order to prevent the development of oral dysbiosis and periodontal pathologies.

### 3.2. Probiotics, Prebiotics and Antibiotics

The use of probiotics and prebiotics is continuously increasing for modulating the GM. Probiotics are a conundrum of beneficial microbes, mostly *Lactobacillus, Bifidobacterium, Lactococcus* and *Saccharomyces*, which can improve several aspects of human physiology, including the GM, immunological parameters and gastrointestinal physiology [70]. Prebiotics are non-digestible dietary fibers and oligosaccharides that selectively nourish beneficial microorganisms, which in turn produce beneficial molecules, such as SCFAs, that can decrease other metabolite toxicity and improve cardiovascular health [71].

The role of probiotics and prebiotics in CVD has been evaluated in a handful of studies. In specific, Malik et al. [72], evaluating the effect of probiotics (*Lactobacillus plantarum 299v*) in individuals with CAD, described that in those individuals there were significant changes in brachial flow-mediated dilation, endothelium-dependent vasodilation and interleukin-8 and 12 levels, without any changes in lipid and trimethylamine oxide levels, thus indicating significant improvement in arterial physiology and inflammation. Moreover, Moludi et al., showed that administration of probiotics (*Lactobacillus Rhamnosus G*) and prebiotics (inulin), in patients with CAD, resulted in significantly decreased levels of C-reactive protein (CRP), LPS and tumor necrosis factor (TNF)-a, in comparison to controls [73]. Another study by the same group, also in patients with CAD using only probiotics (*Lactobacillus Rhamnosus G*), reported a significant decrease in inflammatory markers and LPS, while they have also shown that in patients with myocardial infarction, administration of probiotics is associated with improved echocardiographic indices, compared to baseline, as a result of a positive cardiac remodelling [74]. Moreover, similar protective effects were found in patients with diabetes, where the use of probiotics lowered blood pressure, without, however, any differences in antioxidant markers [75]. Regarding prebiotics, animal heart failure models show an improvement of gut dysbiosis with their use, as well as reduction of endotoxemia [76]. The combination of prebiotics and probiotics is more common in clinical trials, with results showing decreased levels of CRP, nitric oxide and cholesterol levels in CAD [77,78]. Interestingly, several trials also indicate that the combination of those two treatments results in better outcomes in terms of inflammatory marker reduction and reduction of gut permeability, compared to using only one therapy, either probiotic or prebiotic [73,79]. The aforementioned studies provide significant insight regarding the beneficial role of pre-and probiotics in patients with CVD, especially in regard to inflammation. The anti-inflammatory effects of these regimens, along with the preservation or restoration of normal gut flora, could be of great use in patients with both CVD and VHD. However, subsequent trials further documenting their role in influencing clinical outcomes are needed in order to fully understand their role in VHD management and prevention.

Finally, some studies have assessed the effect of GM alteration with antibiotics in the gut–heart relationship. More specifically, a study by Awoyemi et al., evaluated the effect of rifaximin, probiotic yeast *Saccharomyces boulardii* and standard of care, in a 1:1:1 randomized fashion, in patients with heart failure. This trial showed that the administration of the antibiotic or probiotic did not have a significant difference from the standard of care on left ventricular ejection fraction, microbiota diversity TMAO or other inflammation indices [80]. Despite these negative results. Other investigators have found an association of antibiotic use with heart failure risk, potentially in a dose-dependent manner [81]. Thus, there is still need for further research in this topic, in order to fully understand the effect of antibiotics, in association with the GM, in patients with CVD and VHD.

### 3.3. Fecal Microbiota Transplantation (FMT)

Fecal microbiota transplantation (FMT) is an interventional treatment choice for altering an individual’s GM. FMT consists of the administration of a fecal solution from a donor directly into the recipient’s gastrointestinal tract, in order to change their gut microbial composition, which could potentially lead to benefits resulting from an enhanced microbial balance [82]. Most experience with FMT is gained due to its usability in pseudomembranous colitis, with positive results regarding rate of recurrence [83]. Promising results have also been identified in inflammatory bowel disease, with clear short-term benefit but uncertain long-term efficacy and safety [84]. Studies evaluating FMT in CVD are limited; however, it has been shown that FMT with a choline-diet induced TMAO production and an atherosclerosis-prone microbe can transfer atherosclerotic susceptibility in healthy individuals [85], therefore indicating a possible relationship between specific FMT microbes and a higher risk for CVD. However, FMT with atherosclerosis or CVD-resistant microbes, i.e., increasing those microbes with beneficial attributes and reducing those that promote inflammation and CVD, has not been tested yet. The association of FMT with CVD and the positive outcomes in other pathologies is a promising topic of research, where personalized FMT could alter normal gut flora and reduce total CVD risk.

### 3.4. Targeting Microbial Enzymes

Using directed inhibitors of enzymes that produce the previously discussed harmful molecules, such as TMAO, could be beneficial, as it would reduce their levels irrespective of other factors, such as diet. 3,3,Dimethyl-1-Butanol (DMB) is a structural choline analogue which inhibits the microbial synthesis of TMAO [86]. The use of DMB in rats with HF and MI resulted in suppression of TMAO plasma levels and improvement of cardiac function, possibly by inhibiting intracardiac interaction with inflammatory mediators, such as interleukin-8 [86]. Further studies also showcased that DMB, in overload-induced HF mice, can attenuate the development of cardiac remodelling, potentially through inhibition of the NF-κB pathway [87], as well as renal injury [88], aortic stiffening [89] amelioration and endothelial dysfunction prevention [89]. The use of DMB as a therapeutic intervention still requires human trials, which would show the extent of benefit in patients with or at high risk for both CVD and VHD. Finally, other enzymes, such as those mediating the production of SCFAs, should also be targeted by researchers, as increasing their production could lead to significant anti-inflammatory properties and health benefits.

## 4. Future Directions

It is well understood that the pathophysiological connection between the GM and VHD is complex and warrants more research; however, there are several links pointing towards inflammation and inflammation-related heart damage. As studies show, especially in regard to calcified valvular disease, the GM may have a causal relationship as a risk factor for its development. However, there are still limited data to support this novel hypothesis, mostly with the limited number of patients enrolled. Thus, future studies should aim to further evaluate the role of the GM and its effect in VHD development and progression, especially in less studied valves, such as the mitral valve and mitral annulus. Even though specimen studies, where an analysis of the microbes present in the calcified valve would be welcome, their increased complexity and the well-known, expected hurdles in study execution, can shift the attention towards identifying markers of microbial activity, such as TMAO and choline, which would not only reveal a pathogenetic relationship, but also serve as prognosticators of disease severity. Research on non-traditional molecules that could delineate the relationship between the gut microbiome and CVD should also be a frontier of more extensive research. In particular, catestatin, which is a neuroendocrine hormone (chromogranin A derivative) also found in enteroendocrine cells, has a close relationship with the regulation of the GM in preclinical models [90] and has been found to correlate with all-cause death and unplanned heart failure hospitalization in patients with heart failure with reduced ejection fraction (HFrEF) [91]. Further research regarding the relationship of the GM, enteroendocrine cells, CVD and especially VHD is necessary, as it could lead to the identification of novel biomarkers that could link GM dysbiosis and cardiovascular clinical outcomes. Moreover, more focus on interventions that modulate SCFAs and LPS interference with the host, such as diet interventions that increase SCFAs or pre/probiotic combinations that alter the expression of harmful LPSs could add more therapeutic options, on top of targeting the well-studied TMAO. Novel agents such as phenyacetylglutamine, that are currently being more studied, could also reveal more insights into inflammation-independent mechanistic effects that will improve our pathogenesis understanding and potentially lead the efforts for novel targeted therapeutic interventions. Furthermore, genome analysis and identification of individual patterns of GM flora related to VHD could also assist in recognizing early individuals at risk, using screening programs, and intervening early, either by eliminating concomitant CVD risk factors or treating the GM dysbiosis, as aforementioned. Finally, more research is needed regarding treatment alternatives. Currently, there is no evidence regarding potential treatment options and GM alteration in patients with VHD. Further research should identify if intervening with both non-pharmacological and pharmacological measures could alter the course of VHD. However, it should be noted that altering the gut microbiome is a chronic process, where the valve apparatus has already been exposed in harmful interplays and, consequently, the damage in the valve has already been initiated. Thus, it is important to mention that GM alterations with medical interventions would require time (months or years) to take place, while not only one intervention would be needed, but a synergistic effect of aforementioned interventions (diet and pro/prebiotics) would be required in order to observe any results in improving gut dysbiosis. Finally, it is well understood that, as AS becomes more prevalent, a large number of patients will undergo aortic valve replacement, either transcatheter or surgical [92]. Given that prosthetic valve dysfunction is also an increasingly prevalent problem, it would be interesting to investigate if, similarly to native AS, the GM has a role in leaflet calcification and local chronic inflammation. The identification of a link could help physicians better understand the pathophysiology behind prosthetic valve dysfunction, as well as promote preventive measures in order to diminish its effect on disease progression.

## 5. Conclusions

Growing evidence establishes the pathogenetic role of the GM in VHD. The subclinical, chronic inflammation promoted by gut dysbiosis and by microbial mediators such as TMAO and LPS predominantly influences cardiovascular physiology and results in local valvular inflammatory cell infiltration, calcification and cardiac remodelling. GM-modulating agents, especially diet and pro/prebiotics and antibiotics, hold promise for use in maintaining a normal gut flora and ameliorating the harmful effects of gut dysbiosis; however, further exploring the interplay between the gut and the heart and identifying novel therapeutic options is necessary, in order to provide effective prevention and potentially alter the course of VHD.

## Figures and Tables

**Table 1:** Key studies evaluating the role of gut microbiome in valvular heart disease.

Study	Year	Study Type	Participants’ Characteristics (n)	Main Outcomes
Curini et al. [39]	2023	Clinical study	Patients with severe symptomatic calcific AS (n = 20)	T-Cells, and especially T-helper cells, infiltrate calcific AS. The number of CD8+ cells was greater in German patients. German AVs had higher levels of several microbes linked with CVD.
Kocyigit et al. [41]	2020	Clinical study	Patients with severe or moderate AS (n = 60) and controls (n = 48)	Patients with AS had higher choline levels compared to aortic sclerosis patients and controls. TMAO and betaine levels were not significantly different. Choline levels were associated with aortic peak flow velocity and significantly increased in AV with lymphocyte infiltration, osseous metaplasia and calcification.
Jing et al. [42]	2023	Mendelian Randomization study	Patients with exposure to choline, carnitine and PC (114,999; 7997 and 114,999; respectively)	Elevated choline level had a causal relationship with VHD and MI.
Guo et al. [43]	2023	Clinical study	Patients with AS (n = 299) and without AS (n = 711)	TMAO levels were significantly higher in patients with AS, with sustained significant results after baseline characteristics adjustment. Higher TMAO level was associated with significantly higher 2-year all-cause mortality and higher late cumulative mortality.
Xiong et al. [44]	2023	In vitro study	Human AV interstitial cells (AVICs), isolated from AVs	Pathological valves had greater levels of fibrotic molecules (ATF-4, XBP-1, collagen and TGF-β1). This activation was enhanced after stimulation of the cells with TMAO.
Liu et al. [45]	2019	Clinical Study	Individuals with AD, CAD and controls (n = 119)	The bacteria groups for CAD and VHD largely differ. Based on correlation analysis, *Prevotella copri* and *Collinsella aerofaciens* may be of key importance in VHD and CAD, respectively.

Abbreviations: AS: aortic stenosis, AV: aortic valve; CVD: cardiovascular disease; PC: phosphatidylcholine; TMAO: trimethylamine N-oxide.

## References

[1] Amini M., Zayeri F., Salehi M. (2021). Trend analysis of cardiovascular disease mortality, incidence, and mortality-to-incidence ratio: Results from global burden of disease study 2017. BMC Public Health.

[2] Karlsson F.H., Fåk F., Nookaew I., Tremaroli V., Fagerberg B., Petranovic D., Bäckhed F., Nielsen J. (2012). Symptomatic atherosclerosis is associated with an altered gut metagenome. Nat. Commun..

[3] Yamashiro K., Tanaka R., Urabe T., Ueno Y., Yamashiro Y., Nomoto K., Takahashi T., Tsuji H., Asahara T., Hattori N. (2017). Gut dysbiosis is associated with metabolism and systemic inflammation in patients with ischemic stroke. PLoS ONE.

[4] Thursby E., Juge N. (2017). Introduction to the human gut microbiota. Biochem. J..

[5] Minty M., Canceil T., Serino M., Burcelin R., Tercé F., Blasco-Baque V. (2019). Oral microbiota-induced periodontitis: A new risk factor of metabolic diseases. Rev. Endocr. Metab. Disord..

[6] Hugon P., Dufour J.C., Colson P., Fournier P.E., Sallah K., Raoult D. (2015). A comprehensive repertoire of prokaryotic species identified in human beings. Lancet Infect. Dis..

[7] Panzer J.J., Romero R., Greenberg J.M., Winters A.D., Galaz J., Gomez-Lopez N., Theis K.R. (2023). Is there a placental microbiota? A critical review and re-analysis of published placental microbiota datasets. BMC Microbiol..

[8] Stinson L., Hallingström M., Barman M., Viklund F., Keelan J., Kacerovsky M., Payne M., Jacobsson B. (2020). Comparison of Bacterial DNA Profiles in Mid-Trimester Amniotic Fluid Samples From Preterm and Term Deliveries. Front. Microbiol..

[9] Miko E., Csaszar A., Bodis J., Kovacs K. (2022). The Maternal–Fetal Gut Microbiota Axis: Physiological Changes, Dietary Influence, and Modulation Possibilities. Life.

[10] Rodríguez J.M., Murphy K., Stanton C., Ross R.P., Kober O.I., Juge N., Avershina E., Rudi K., Narbad A., Jenmalm M.C. (2015). The composition of the gut microbiota throughout life, with an emphasis on early life. Microb. Ecol. Health Dis..

[11] Jandhyala S.M. (2015). Role of the normal gut microbiota. World J. Gastroenterol..

[12] Corrêa-Oliveira R., Fachi J.L., Vieira A., Sato F.T., Vinolo M.A.R. (2016). Regulation of immune cell function by short-chain fatty acids. Clin. Transl. Immunol..

[13] Morrison D.J., Preston T. (2016). Formation of short chain fatty acids by the gut microbiota and their impact on human metabolism. Gut Microbes.

[14] Natividad J.M.M., Verdu E.F. (2013). Modulation of intestinal barrier by intestinal microbiota: Pathological and therapeutic implications. Pharmacol. Res..

[15] Telesford K.M., Yan W., Ochoa-Reparaz J., Pant A., Kircher C., Christy M.A., Begum-Haque S., Kasper D.L., Kasper L.H. (2015). A commensal symbiotic factor derived from *Bacteroides fragilis* promotes human CD39^+^ Foxp3^+^ T cells and T_reg_ function. Gut Microbes.

[16] Mousa W.K., Chehadeh F., Husband S. (2022). Microbial dysbiosis in the gut drives systemic autoimmune diseases. Front. Immunol..

[17] Su X., Zhao Y., Li Y., Ma S., Wang Z. (2020). Gut dysbiosis is associated with primary hypothyroidism with interaction on gut-thyroid axis. Clin. Sci..

[18] Din A.U., Mazhar M., Waseem M., Ahmad W., Bibi A., Hassan A., Ali N., Gang W., Qian G., Ullah R. (2021). SARS-CoV-2 microbiome dysbiosis linked disorders and possible probiotics role. Biomed. Pharmacother..

[19] Lau K., Srivatsav V., Rizwan A., Nashed A., Liu R., Shen R., Akhtar M. (2017). Bridging the Gap between Gut Microbial Dysbiosis and Cardiovascular Diseases. Nutrients.

[20] Kim M., Lee S.W., Kim J., Shin Y., Chang F., Kim J.M., Cong X., Yu G.Y., Park K. (2021). LPS-induced epithelial barrier disruption via hyperactivation of CACC and ENaC. Am. J. Physiol. Cell Physiol..

[21] Kim S., Goel R., Kumar A., Qi Y., Lobaton G., Hosaka K., Mohammed M., Handberg E.M., Richards E.M., Pepine C.J. (2018). Imbalance of gut microbiome and intestinal epithelial barrier dysfunction in patients with high blood pressure. Clin. Sci..

[22] Violi F., Cammisotto V., Bartimoccia S., Pignatelli P., Carnevale R., Nocella C. (2023). Gut-derived low-grade endotoxaemia, atherothrombosis and cardiovascular disease. Nat. Rev. Cardiol..

[23] Suzuki K., Ohkuma M., Nagaoka I. (2019). Bacterial lipopolysaccharide and antimicrobial LL-37 enhance ICAM-1 expression and NF-κB p65 phosphorylation in senescent endothelial cells. Int. J. Mol. Med..

[24] Gorabi A.M., Kiaie N., Khosrojerdi A., Jamialahmadi T., Al-Rasadi K., Johnston T.P., Sahebkar A. (2022). Implications for the role of lipopolysaccharide in the development of atherosclerosis. Trends Cardiovasc. Med..

[25] Pastori D., Carnevale R., Nocella C., Novo M., Santulli M., Cammisotto V., Menichelli D., Pignatelli P., Violi F. (2017). Gut-Derived Serum Lipopolysaccharide is Associated With Enhanced Risk of Major Adverse Cardiovascular Events in Atrial Fibrillation: Effect of Adherence to Mediterranean Diet. J. Am. Heart Assoc..

[26] Krüger S., Kunz D., Graf J., Stickel T., Merx M.W., Koch K.C., Janssens U., Hanrath P. (2007). Endotoxin hypersensitivity in chronic heart failure. Int. J. Cardiol..

[27] Ding Y., Subramanian S., Montes V.N., Goodspeed L., Wang S., Han C., Teresa A.S., Kim J., O’Brien K.D., Chait A. (2012). Toll-Like Receptor 4 Deficiency Decreases Atherosclerosis but Does Not Protect Against Inflammation in Obese Low-Density Lipoprotein Receptor–Deficient Mice. Arterioscler. Thromb. Vasc. Biol..

[28] Seldin M.M., Meng Y., Qi H., Zhu W., Wang Z., Hazen S.L., Lusis A.J., Shih D.M. (2016). Trimethylamine N-Oxide Promotes Vascular Inflammation Through Signaling of Mitogen-Activated Protein Kinase and Nuclear Factor-κB. J. Am. Heart Assoc..

[29] Jing L., Zhang H., Xiang Q., Shen L., Guo X., Zhai C., Hu H. (2022). Targeting Trimethylamine N-Oxide: A New Therapeutic Strategy for Alleviating Atherosclerosis. Front. Cardiovasc. Med..

[30] Xie Z., Liu X., Huang X., Liu Q., Yang M., Huang D., Zhao P., Tian J., Wang X., Hou J. (2021). Remodelling of gut microbiota by Berberine attenuates trimethylamine N-oxide-induced platelet hyperreaction and thrombus formation. Eur. J. Pharmacol..

[31] Zong X., Fan Q., Yang Q., Pan R., Zhuang L., Xi R., Zhang R., Tao R. (2022). Trimethyllysine, a trimethylamine N-oxide precursor, predicts the presence, severity, and prognosis of heart failure. Front. Cardiovasc. Med..

[32] Yang W., Zhang S., Zhu J., Jiang H., Jia D., Ou T., Qi Z., Zou Y., Qian J., Sun A. (2019). Gut microbe-derived metabolite trimethylamine N-oxide accelerates fibroblast-myofibroblast differentiation and induces cardiac fibrosis. J. Mol. Cell Cardiol..

[33] Organ C.L., Otsuka H., Bhushan S., Wang Z., Bradley J., Trivedi R., Polhemus D.J., Tang W.H., Wu Y., Hazen S.L. (2016). Choline Diet and Its Gut Microbe–Derived Metabolite, Trimethylamine N-Oxide, Exacerbate Pressure Overload–Induced Heart Failure. Circ. Heart Fail..

[34] Organ C.L., Li Z., Sharp T.E., Polhemus D.J., Gupta N., Goodchild T.T., Tang W.H.W., Hazen S.L., Lefer D.J. (2020). Nonlethal Inhibition of Gut Microbial Trimethylamine N-oxide Production Improves Cardiac Function and Remodeling in a Murine Model of Heart Failure. J. Am. Heart Assoc..

[35] Gupta N., Buffa J.A., Roberts A.B., Sangwan N., Skye S.M., Li L., Ho K.J., Varga J., DiDonato J.A., Tang W.H.W. (2020). Targeted Inhibition of Gut Microbial Trimethylamine N-Oxide Production Reduces Renal Tubulointerstitial Fibrosis and Functional Impairment in a Murine Model of Chronic Kidney Disease. Arterioscler. Thromb. Vasc. Biol..

[36] Zhao P., Zhao S., Tian J., Liu X. (2022). Significance of Gut Microbiota and Short-Chain Fatty Acids in Heart Failure. Nutrients.

[37] Liu Y., Liu S., Zhao Z., Song X., Qu H., Liu H. (2021). Phenylacetylglutamine is associated with the degree of coronary atherosclerotic severity assessed by coronary computed tomographic angiography in patients with suspected coronary artery disease. Atherosclerosis.

[38] Abdul-Rahman T., Lizano-Jubert I., Garg N., Talukder S., Lopez P.P., Awuah W.A., Shah R., Chambergo D., Cantu-Herrera E., Farooqi M. (2023). The common pathobiology between coronary artery disease and calcific aortic stenosis: Evidence and clinical implications. Prog. Cardiovasc. Dis..

[39] Curini L., Alushi B., Christopher M.R., Baldi S., Di Gloria L., Stefano P., Laganà A., Iannone L., Grubitzsch H., Landmesser U. (2023). The first taxonomic and functional characterization of human CAVD-associated microbiota. Microb. Cell.

[40] Raddatz M.A., Madhur M.S., Merryman W.D. (2019). Adaptive immune cells in calcific aortic valve disease. Am. J. Physiol. Heart Circ. Physiol..

[41] Kocyigit D., Tokgozoglu L., Gurses K.M., Stahlman M., Boren J., Soyal M.F.T., Canpınar H., Guc D., Saglam Ayhan A., Hazirolan T. (2021). Association of dietary and gut microbiota-related metabolites with calcific aortic stenosis. Acta Cardiol..

[42] Jing W., Huang S., Xiang P., Huang J., Yu H. (2023). Dietary precursors and cardiovascular disease: A Mendelian randomization study. Front. Cardiovasc. Med..

[43] Guo Y., Xu S., Zhan H., Chen H., Hu P., Zhou D., Dai H., Liu X., Hu W., Zhu G. (2023). Trimethylamine N-Oxide Levels Are Associated with Severe Aortic Stenosis and Predict Long-Term Adverse Outcome. J. Clin. Med..

[44] Xiong Z., Li J., Huang R., Zhou H., Xu X., Zhang S., Xie P., Li M., Guo Y., Liao X. (2024). The gut microbe-derived metabolite trimethylamine-N-oxide induces aortic valve fibrosis via PERK/ATF-4 and IRE-1α/XBP-1s signaling in vitro and in vivo. Atherosclerosis.

[45] Liu Z., Li J., Liu H., Tang Y., Zhan Q., Lai W., Ao L., Meng X., Ren H., Xu D. (2019). The intestinal microbiota associated with cardiac valve calcification differs from that of coronary artery disease. Atherosclerosis.

[46] Sanz M., Marco del Castillo A., Jepsen S., Gonzalez-Juanatey J.R., D’Aiuto F., Bouchard P., Chapple I., Dietrich T., Gotsman I., Graziani F. (2020). Periodontitis and cardiovascular diseases: Consensus report. J. Clin. Periodontol..

[47] Zardawi F., Gul S., Abdulkareem A., Sha A., Yates J. (2021). Association Between Periodontal Disease and Atherosclerotic Cardiovascular Diseases: Revisited. Front. Cardiovasc. Med..

[48] Haraszthy V.I., Zambon J.J., Trevisan M., Zeid M., Genco R.J. (2000). Identification of Periodontal Pathogens in Atheromatous Plaques. J. Periodontol..

[49] Xie M., Tang Q., Nie J., Zhang C., Zhou X., Yu S., Sun J., Cheng X., Dong N., Hu Y. (2020). BMAL1-Downregulation Aggravates *Porphyromonas Gingivalis*-Induced Atherosclerosis by Encouraging Oxidative Stress. Circ. Res..

[50] Seoane T., Bullon B., Fernandez-Riejos P., Garcia-Rubira J.C., Garcia-Gonzalez N., Villar-Calle P., Quiles J.L., Battino M., Bullon P. (2022). Periodontitis and Other Risk Factors Related to Myocardial Infarction and Its Follow-Up. J. Clin. Med..

[51] Nakano K., Nemoto H., Nomura R., Inaba H., Yoshioka H., Taniguchi K., Amano A., Ooshima T. (2009). Detection of oral bacteria in cardiovascular specimens. Oral. Microbiol. Immunol..

[52] Oliveira F.A.F., Forte C.P.F., Silva P.G.d.B., Lopes C.B., Montenegro R.C., Santos Â.K.C.R.D., Sobrinho C.R.M.R., Mota M.R.L., Sousa F.B., Alves A.P.N.N. (2015). Molecular Analysis of Oral Bacteria in Heart Valve of Patients With Cardiovascular Disease by Real-Time Polymerase Chain Reaction. Medicine.

[53] Sia S., Jan M., Wang Y., Huang Y., Wei J.C. (2021). Periodontitis is associated with incidental valvular heart disease: A nationwide population-based cohort study. J. Clin. Periodontol..

[54] Aardema H., Lisotto P., Kurilshikov A., Diepeveen J.R.J., Friedrich A.W., Sinha B., de Smet A.M.G.A., Harmsen H.J.M. (2020). Marked Changes in Gut Microbiota in Cardio-Surgical Intensive Care Patients: A Longitudinal Cohort Study. Front. Cell Infect. Microbiol..

[55] Xue L., Ding Y., Qin Q., Liu L., Ding X., Zhou Y., Liu K., Singla R.K., Shen K., Din A.U. (2023). Assessment of the impact of intravenous antibiotics treatment on gut microbiota in patients: Clinical data from pre-and post-cardiac surgery. Front. Cell Infect. Microbiol..

[56] Karl J.P., Fu X., Wang X., Zhao Y., Shen J., Zhang C., Wolfe B.E., Saltzman E., Zhao L., Booth S.L. (2015). Fecal menaquinone profiles of overweight adults are associated with gut microbiota composition during a gut microbiota–targeted dietary intervention. Am. J. Clin. Nutr..

[57] Chen W., Qian J., Fu J., Wu T., Lv M., Jiang S., Zhang J. (2022). Changes in the Gut Microbiota May Affect the Clinical Efficacy of Oral Anticoagulants. Front. Pharmacol..

[58] David L.A., Maurice C.F., Carmody R.N., Gootenberg D.B., Button J.E., Wolfe B.E., Ling A.V., Devlin A.S., Varma Y., Fischbach M.A. (2014). Diet rapidly and reproducibly alters the human gut microbiome. Nature.

[59] Cani P.D., Bibiloni R., Knauf C., Waget A., Neyrinck A.M., Delzenne N.M., Burcelin R. (2008). Changes in Gut Microbiota Control Metabolic Endotoxemia-Induced Inflammation in High-Fat Diet–Induced Obesity and Diabetes in Mice. Diabetes.

[60] Agans R., Gordon A., Kramer D.L., Perez-Burillo S., Rufián-Henares J.A., Paliy O. (2018). Dietary Fatty Acids Sustain the Growth of the Human Gut Microbiota. Appl. Environ. Microbiol..

[61] Makki K., Deehan E.C., Walter J., Bäckhed F. (2018). The Impact of Dietary Fiber on Gut Microbiota in Host Health and Disease. Cell Host Microbe.

[62] Barber T.M., Kabisch S., Pfeiffer A.F.H., Weickert M.O. (2023). The Effects of the Mediterranean Diet on Health and Gut Microbiota. Nutrients.

[63] Merra G., Noce A., Marrone G., Cintoni M., Tarsitano M.G., Capacci A., De Lorenzo A. (2020). Influence of Mediterranean Diet on Human Gut Microbiota. Nutrients.

[64] Delgado-Lista J., Alcala-Diaz J.F., Torres-Peña J.D., Quintana-Navarro G.M., Fuentes F., Garcia-Rios A., Ortiz-Morales A.M., Gonzalez-Requero A.I., Perez-Caballero A.I., Yubero-Serrano E.M. (2022). Long-term secondary prevention of cardiovascular disease with a Mediterranean diet and a low-fat diet (CORDIOPREV): A randomised controlled trial. Lancet.

[65] Kusy K., Błażejewski J., Gilewski W., Karasek D., Banach J., Bujak R., Zieliński J., Sinkiewicz W., Grześk G. (2021). Aging Athlete’s Heart: An Echocardiographic Evaluation of Competitive Sprint- versus Endurance-Trained Master Athletes. J. Am. Soc. Echocardiogr..

[66] Martinon P., Fraticelli L., Giboreau A., Dussart C., Bourgeois D., Carrouel F. (2021). Nutrition as a Key Modifiable Factor for Periodontitis and Main Chronic Diseases. J. Clin. Med..

[67] Woelber J.P., Bremer K., Vach K., König D., Hellwig E., Ratka-Krüger P., Al-Ahmad A., Tennert C. (2017). An oral health optimized diet can reduce gingival and periodontal inflammation in humans—A randomized controlled pilot study. BMC Oral Health.

[68] Altun E., Walther C., Borof K., Petersen E., Lieske B., Kasapoudis D., Jalilvand N., Beikler T., Jagemann B., Zyriax B.C. (2021). Association between Dietary Pattern and Periodontitis—A Cross-Sectional Study. Nutrients.

[69] Lertpimonchai A., Rattanasiri S., Arj-Ong Vallibhakara S., Attia J., Thakkinstian A. (2017). The association between oral hygiene and periodontitis: A systematic review and meta-analysis. Int. Dent. J..

[70] Khalesi S., Bellissimo N., Vandelanotte C., Williams S., Stanley D., Irwin C. (2019). A review of probiotic supplementation in healthy adults: Helpful or hype?. Eur. J. Clin. Nutr..

[71] Pandey K.R., Naik S.R., Vakil B.V. (2015). Probiotics, prebiotics and synbiotics—A review. J. Food Sci. Technol..

[72] Malik M., Suboc T.M., Tyagi S., Salzman N., Wang J., Ying R., Tanner M.J., Kakarla M., Baker J.E., Widlansky M.E. (2018). *Lactobacillus plantarum* 299v Supplementation Improves Vascular Endothelial Function and Reduces Inflammatory Biomarkers in Men With Stable Coronary Artery Disease. Circ. Res..

[73] Moludi J., Khedmatgozar H., Nachvak S.M., Abdollahzad H., Moradinazar M., Tabaei A.S. (2022). The effects of co-administration of probiotics and prebiotics on chronic inflammation, and depression symptoms in patients with coronary artery diseases: A randomized clinical trial. Nutr. Neurosci..

[74] Moludi J., Saiedi S., Ebrahimi B., Alizadeh M., Khajebishak Y., Ghadimi S.S. (2021). Probiotics Supplementation on Cardiac Remodeling Following Myocardial Infarction: A Single-Center Double-Blind Clinical Study. J. Cardiovasc. Transl. Res..

[75] Ahmadian F., Razmpoosh E., Ejtahed H.S., Javadi M., Mirmiran P., Azizi F. (2022). Effects of probiotic supplementation on major cardiovascular-related parameters in patients with type-2 diabetes mellitus: A secondary-data analysis of a randomized double-blind controlled trial. Diabetol. Metab. Syndr..

[76] Vlasov A.A., Shperling M.I., Terkin D.A., Bystrova O.V., Osipov G.A., Salikova S.P., Grinevich V.B. (2020). Effect of Prebiotic Complex on Gut Microbiota and Endotoxemia in Female Rats with Modeled Heart Failure. Bull. Exp. Biol. Med..

[77] Farrokhian A., Raygan F., Soltani A., Tajabadi-Ebrahimi M., Sharifi Esfahani M., Karami A.A., Asemi Z. (2019). The Effects of Synbiotic Supplementation on Carotid Intima-Media Thickness, Biomarkers of Inflammation, and Oxidative Stress in People with Overweight, Diabetes, and Coronary Heart Disease: A Randomized, Double-Blind, Placebo-Controlled Trial. Probiotics Antimicrob. Proteins.

[78] Tajabadi-Ebrahimi M., Sharifi N., Farrokhian A., Raygan F., Karamali F., Razzaghi R., Taheri S., Asemi Z. (2017). A Randomized Controlled Clinical Trial Investigating the Effect of Synbiotic Administration on Markers of Insulin Metabolism and Lipid Profiles in Overweight Type 2 Diabetic Patients with Coronary Heart Disease. Exp. Clin. Endocrinol. Diabetes.

[79] Liu M., Tandorost A., Moludi J., Dey P. (2024). Prebiotics Plus Probiotics May Favorably Impact on Gut Permeability, Endocannabinoid Receptors, and Inflammatory Biomarkers in Patients with Coronary Artery Diseases: A Clinical Trial. Food Sci. Nutr..

[80] Awoyemi A., Mayerhofer C., Felix A.S., Hov J.R., Moscavitch S.D., Lappegård K.T., Hovland A., Halvorsen S., Halvorsen B., Gregersen I. (2021). Rifaximin or Saccharomyces boulardii in heart failure with reduced ejection fraction: Results from the randomized GutHeart trial. EBioMedicine.

[81] Loosen S.H., Krieg S., Gaensbacher J., Doege C., Krieg A., Luedde T., Luedde M., Roderburg C., Kostev K. (2023). The Association between Antibiotic Use and the Incidence of Heart Failure: A Retrospective Case-Control Study of 162,188 Outpatients. Biomedicines.

[82] Gupta S., Allen-Vercoe E., Petrof E.O. (2016). Fecal microbiota transplantation: In perspective. Ther. Adv. Gastroenterol..

[83] Van Nood E., Vrieze A., Nieuwdorp M., Fuentes S., Zoetendal E.G., de Vos W.M., Visser C.E., Kuijper E.J., Bartelsman J.F., Tijssen J.G. (2013). Duodenal Infusion of Donor Feces for Recurrent *Clostridium difficile*. N. Engl. J. Med..

[84] Paramsothy S., Paramsothy R., Rubin D.T., Kamm M.A., Kaakoush N.O., Mitchell H.M., Castaño-Rodríguez N. (2017). Faecal Microbiota Transplantation for Inflammatory Bowel Disease: A Systematic Review and Meta-analysis. J. Crohn’s Colitis.

[85] Gregory J.C., Buffa J.A., Org E., Wang Z., Levison B.S., Zhu W., Wagner M.A., Bennett B.J., Li L., DiDonato J.A. (2015). Transmission of Atherosclerosis Susceptibility with Gut Microbial Transplantation. J. Biol. Chem..

[86] Li X., Sun Y., Zhang X., Wang J. (2019). Reductions in gut microbiota-derived metabolite trimethylamine N-oxide in the circulation may ameliorate myocardial infarction-induced heart failure in rats, possibly by inhibiting interleukin-8 secretion. Mol. Med. Rep..

[87] Wang G., Kong B., Shuai W., Fu H., Jiang X., Huang H. (2020). 3,3-Dimethyl-1-butanol attenuates cardiac remodeling in pressure-overload-induced heart failure mice. J. Nutr. Biochem..

[88] Zou D., Li Y., Sun G. (2021). Attenuation of Circulating Trimethylamine N-Oxide Prevents the Progression of Cardiac and Renal Dysfunction in a Rat Model of Chronic Cardiorenal Syndrome. Front. Pharmacol..

[89] Casso A.G., VanDongen N.S., Gioscia-Ryan R.A., Clayton Z.S., Greenberg N.T., Ziemba B.P., Hutton D.A., Neilson A.P., Davy K.P., Seals D.R. (2022). Initiation of 3,3-dimethyl-1-butanol at midlife prevents endothelial dysfunction and attenuates in vivo aortic stiffening with ageing in mice. J. Physiol..

[90] González-Dávila P., Schwalbe M., Danewalia A., Dalile B., Verbeke K., Mahata S.K., El Aidy S. (2022). Catestatin selects for colonization of antimicrobial-resistant gut bacterial communities. ISME J..

[91] Wołowiec Ł., Banach J., Budzyński J., Wołowiec A., Kozakiewicz M., Bieliński M., Jaśniak A., Olejarczyk A., Grześk G. (2023). Prognostic Value of Plasma Catestatin Concentration in Patients with Heart Failure with Reduced Ejection Fraction in Two-Year Follow-Up. J. Clin. Med..

[92] Carroll J.D., Mack M.J., Vemulapalli S., Herrmann H.C., Gleason T.G., Hanzel G., Deeb G.M., Thourani V.H., Cohen D.J., Desai N. (2021). STS-ACC TVT Registry of Transcatheter Aortic Valve Replacement. Ann. Thorac. Surg..

